# Effects of Tele-Rehabilitation Compared with Home-Based in-Person Rehabilitation for Older Adult’s Function after Hip Fracture

**DOI:** 10.3390/ijerph18105493

**Published:** 2021-05-20

**Authors:** Mariana Ortiz-Piña, Pablo Molina-Garcia, Pedro Femia, Maureen C. Ashe, Lydia Martín-Martín, Susana Salazar-Graván, Zeus Salas-Fariña, Rafael Prieto-Moreno, Yolanda Castellote-Caballero, Fernando Estevez-Lopez, Patrocinio Ariza-Vega

**Affiliations:** 1 Department of Physiotherapy, Faculty of Health Science, University of Granada, Avenida de la Investigación, s/n, 18016 Granada, Spain; marianaop@correo.ugr.es (M.O.-P.); lydia@ugr.es (L.M.-M.); pariza@ugr.es (P.A.-V.); 2 Biohealth Research Institute, Physical Medicine and Rehabilitation Service, Virgen de las Nieves University Hospital, Jaén Street, s/n, 18013 Granada, Spain; 3 Department of Statistics and Operational Research, Faculty of Medicine, University of Granada, Avenida de la Investigación, 11, 18016 Granada, Spain; pfemia@ugr.es; 4 Centre for Hip Health and Mobility, Department of Family Practice, University of British Columbia, Vancouver, BC V6T, Canada; maureen.ashe@ubc.ca; 5 Orthopaedic Surgery and Traumatology Service, Virgen de las Nieves University Hospital, Jaén Street, s/n, 18013 Granada, Spain; susanasagr@gmail.com; 6 PROFITH “PROmoting FITness and Health through Physical Activity” Research Group, Department of Physical Education and Sport, Faculty of Sport Sciences, University of Granada, Alfacar Street, 402, 18011 Granada, Spain; zeusmsf@gmail.com; 7 PA-HELP “Physical Activity for HEaLth Promotion” Research Group, University of Granada, Alfacar Street, 402, 18011 Granada, Spain; rafaprieto58@hotmail.com; 8 Department of Physiotherapy, University of Jaén, Lagunillas Univerity Campus, 23009 Jaén, Spain; yolanda@castellote.net; 9 Department of Child and Adolescent Psychiatry/Psychology, Erasmus MC University Medical Center, Postbus 2060, 3000 CB Rotterdam, The Netherlands; fer@estevez-lopez.com

**Keywords:** activities of daily living, mobility, rehabilitation, exercise

## Abstract

This study aimed to examine the effect of a multidisciplinary tele-rehabilitation program on functional recovery of older adults with hip fracture compared with home-based in-person rehabilitation. In this single-blinded, non-randomized clinical trial, we included older with hip fracture. The tele-rehabilitation group received a 12-week tele-rehabilitation program (supervised by their family caregivers). The control group received the usual postoperative rehabilitation provided by the Andalusian health system (Spain). The primary outcome was the patient-reported functional status assessed with the Functional Independence Measure. We also measured performance-based functional recovery using the Timed Up and Go Test and Short Physical Performance Battery. We performed both a per-protocol (62 participants; 28 tele-rehabilitation and 34 control groups) and an intention-to-treat analysis (71 participants; 35 tele-rehabilitation and 36 control groups). Participants who used the tele-rehabilitation program had higher Functional Independence Measure scores (high effect size: 0.98 Cohen’s d; *p* < 0.001) and better performance in the Timed Up and Go Test (medium effect size: 0.63 Cohen’s d; *p* = 0.025) compared with the control group. Differences between groups post-intervention were not statistically significant in the Short Physical Performance Battery. The tele-rehabilitation intervention proposed in this study is a valuable treatment option in the recovery process for older adults with hip fracture. ClinicalTrials.gov Identifier: NCT02968589.

## 1. Introduction

Osteoporosis is a common chronic condition associated with aging and is related to low-trauma or fragility fractures at the wrist, spine, or hip [1]. Hip fracture is the most serious of low trauma fractures due to potential effects on physical and psychological factors producing emotional stress and reduction in functional independence and quality of life that can be maintained even one year after fracture [2,3].

Early rehabilitation in hospital and post-discharge can improve older adults’ functional recovery [4]. Home rehabilitation is an important option post-hospital discharge for hip fracture [5], and given the advances in information and communication technologies (ICT) [6], tele-rehabilitation (tele-rehab) (“the provision of rehabilitation services at a distance”) is another possible delivery mode. There are many studies highlighting the benefits of tele-rehabilitation in other clinical areas, such as post joint arthroplasty [7,8], cancer [9], stroke [10], and/or heart conditions [11]. Conversely, there is a gap in evidence and practice for tele-rehabilitation for older adults with hip fracture [12]. Only a limited number of studies [13,14,15,16,17] used online ICTs (such as videos) to deliver tele-rehabilitation after hip fracture, with noteworthy limitations, such as small sample sizes (between 14 and 40 patients) [13,14,16,17], or no control group [13].

There may be perceived barriers for older adults to access and use ICTs, as the average age for someone with hip fracture is mid-eighties. However, Crotty et al. [18] delivered care remotely via tablets to older adults (mean age (standard deviation) = 73 (10) years), and they suggested that this population could be considered future users of tele-rehab. Likewise, in the systematic review by Cottrell and colleagues, the conclusion was that tele-rehabilitation (for musculoskeletal conditions) is an effective and comparable rehabilitation option—even for older adults [19]. Considering the limited evidence for the effectiveness of tele-rehabilitation after hip fracture, we designed and tested a clinical tele-rehabilitation program called @ctivehip for older adults and their informal (family) caregivers. The aim of this study is to compare the tele-rehabilitation program with home-based in-person rehabilitation delivered by the Andalusian health system for patient-reported and performance-based functional recovery of older adults with hip fracture.

## 2. Materials and Methods

### 2.1. Study Design and Population

This was a single-blinded, non-randomized clinical trial conducted according to the established guidelines by the Helsinki Declaration and Law 14/2007 on Biomedical Research. The non-inferiority design was based on the evidence gap of the effectiveness of tele-rehabilitation programs for patients with a hip fracture on functional recovery [12] versus the clinical and scientific evidence of the benefits of home-based supervised rehabilitation for older adults with hip fracture [5]. We chose a non-randomized controlled design considering the following factors. First, as mentioned, tele-rehabilitation is novel in this population [12], and it requires patients have access to a computer and the Internet. Second, the use of ICTs could make significant demands on some study participants. Third, the following ethical issue was considered: participants who used the tele-rehabilitation program could engage in more rehab sessions at home, although the sessions were supervised by their informal caregivers instead of physiotherapists (PT) and occupational therapists (OT). The option of offering the tele-rehabilitation program to participants in the control group at the end of the study did not solve the ethical problem because the first three months after hip fracture are crucial for the functional recovery of patients [20]. Fourth, we included patients’ preferences [21] in this clinical trial because we aimed to know the real-world implementation for this program in daily clinical routines. The study was approved by the Ethics Committee of the Research Center of Granada (CEI-GRANADA) and registered at ClinicalTrials.gov (Identifier: NCT02968589). Both patients and caregivers signed consent forms.

All consecutive patients admitted with hip fracture who met the following inclusion criteria were invited to enroll in the study: (1) had hip fracture surgery; (2) were 65 years or older; (3) had a high (self-reported) pre-fracture functional level the week before the fracture (Functional Independence Measure (FIM) index > 90 points); (4) could weight-bear at 48 h after surgery; (5) community-dwelling (in own home or with a relative) after hospitalization; and (6) had internet access and/or a family caregiver with access. Exclusion criteria were: (1) presence of severe cognitive impairment (Mini-mental State Examination score [22] lower than 24 points); (2) terminal disease (not expected to live beyond six months); or (3) post-surgery complications, such as revision surgery, and or respiratory or heart problems, that made it impossible to begin rehabilitation during the first week after surgery.

### 2.2. Recruitment, Allocation, and Blinding

Recruitment took place at the University Hospital of Granada between January 2017 and July 2018. There were two acute hospital staff, one OT and one PT, who invited all eligible consecutive patients and caregivers to join the study. Patients and their caregivers were given the choice of allocation to (1) usual care, an educational workshop, and OT and PT delivered home rehab (control group); or (2) usual care, an educational workshop, and the tele-rehabilitation intervention (@ctivehip intervention group). We chose this study design because of the pragmatic nature of our practice-based research, the novelty of the tele-rehabilitation intervention, and the importance of patients’ preferences in health care delivery under real-world settings [21].

It was not possible to blind patients and their caregivers to group allocation. However, data collection was conducted by an OT, PT, and sport scientist who were blinded to group allocation. Data analysis was performed by a statistician, a PT, and an OT, also blinded to group allocation.

### 2.3. Tele-Rehabilitation Characteristics

#### 2.3.1. Both Groups: Usual Care during a Hospital Stay and Caregivers’ Workshop

After hip fracture, usual care consisted of a few sessions of rehabilitation (between 2–5 sessions) during a hospital stay. Caregivers were also invited to participate in one workshop on postoperative patient management and recommendations for home. The workshop was delivered by hospital staff (an OT and a PT) twice a week on the acute care unit at the Virgen de las Nieves University Hospital (Granada, Spain). In addition, an informational leaflet with recommendations and exercises for home was given to patients and caregivers during the hospital stay.

#### 2.3.2. Tele-Rehabilitation Group (@ctivehip)

Participants in the tele-rehabilitation group (patients and caregivers) received usual care during the hospital stay, the invitation to participate in the workshop described above, and a 12-week multidisciplinary tele-rehabilitation program. Details of the program are described elsewhere in the @ctivehip protocol [23], but a summary is below.

The design of the tele-rehabilitation program was based on: (1) a previous published home-based exercise program for patients with hip fracture [24]; (2) exercise and physical activity position stand for older adults from the American Colleague of Sports Medicine [25]; and (3) the clinical experience of a multidisciplinary group composed of sport sciences professionals, physical therapists, occupational therapists, and orthopedic surgeon consultants [23]. The tele-rehabilitation program had two online components: (1) three exercise sessions and (2) two occupational therapy sessions. Each online component had on-demand (pre-recorded) instructional videos and written instructions for activities and exercises appropriate to the patients’ functional status. The difficulty of sessions was categorized into four levels (Beginners, Moderate, Advanced 1, and Advanced 2). The exercise program included lower and upper body strengthening exercises, balance exercises, and cardiovascular exercises. Each session included three warm-up exercises, followed by nine to ten exercises with a minimum of 10 repetitions during the first week to a maximum of 24 repetitions during the last week, and one relaxation exercise at the end of the session. The occupational therapy program included videos describing the safest way to perform activities of daily living, a description of self-care activities and walking aids, and options to create a safer home environment to prevent new falls. Each session was 50–60 min in duration and was supervised by the informal caregivers at home who had the option to request weekly videoconferences with PTs or OTs. The adherence of the tele-rehabilitation group was recorded automatically on the web page at the end of each session.

#### 2.3.3. Control Group

In addition to usual care during the hospital stay and the caregiver workshop, patients in this group also received the usual postoperative home-based in-person rehabilitation delivered by the Andalusian health system (between 5–15 sessions of physiotherapy and occupational therapy).

### 2.4. Primary Outcome: Functional Status

All patients and their caregivers enrolled in the study were assessed at three time points: (1) before hospital discharge; (2) one month after hospital discharge; and (3) three months after hospital discharge (end of the tele-rehabilitation program). We asked participants to prospectively self-report, via the online platform, any adverse or serious adverse events which were reviewed weekly by one OT.

The main outcome measure was patient-reported functional status assessed with the FIM [26] at 3 months. The FIM score reflects the level of assistance a person needs to perform the activities of daily living, considering 18 items grouped into six categories of activity: self-care, sphincter control, mobility, locomotion, communication, and cognition. The total score ranges from 18 to 126 points; higher scores indicate a higher functional level [26]. The pre-fracture FIM score was filled out during the first interview at the hospital, and it was based on the responses of the patients about the performed tasks in the week prior to the hip fracture. The internal consistency of the FIM was excellent, with Cronbach’s α = 0.95 [27].

### 2.5. Secondary Outcome: Physical Performance

We also assessed functional recovery using two performance-based tests: (1) the Timed Up and Go Test (TUG) measures the time that a person takes to perform the following tasks: get up from a chair, walk three meters, turn around, walk back three meters, and sit back down in the chair [28]. Participants were instructed to walk in the most comfortable and safe way possible, in addition to using a rollator at the time of the evaluations. Three tests were performed in each session, and the average of the three measurements was recorded. Internal consistency of the TUG was excellent (Cronbach’s α = 0.97) [29] and (2) the Short Physical Performance Battery (SPPB) [30] consists of three subscales: balance, walking, and chair stands. The score ranges from 0 to 12 points, with higher scores indicating better mobility [30]. The internal consistency of the SPPB was high, with Cronbach´s α = 0.87 [31].

### 2.6. Descriptive Information

Sociodemographic data, such as age, gender, educational level, falls in the previous year, and place of residence, were collected during the interviews with patients and their caregivers. We collected clinical data from medical charts for hospital length of stay, health status (measured by the American Society of Anesthesiologists’ score) [32], 24 h delay of surgery (yes or no), and type of fracture.

### 2.7. Sample Size

The study was designed to have an 80% chance of detecting an 8.7% difference between groups for the primary outcome (FIM) according to data from a previously published home-based rehabilitation intervention on hip fracture patients [33]. We set the alpha error at 5% and used a two-sample t-test. We also considered the minimal clinically significant difference in the FIM index (11 points) between groups at three months [34]. By adding 35% to account for potential losses, based on the study of tele-rehabilitation in patients with hip fracture carried out by Tappen et al. [15], we required 70 patients (35 participants/group) for this study. We used the Epidat 3.1 Software (Xunta of Galicia) for the sample size calculation.

### 2.8. Data Analyses

Before performing the analysis, continuous variables were checked for normal distribution via the visual inspection of histograms together with the Kolmogorov–Smirnov test. All the outcomes demonstrated a non-normal distribution and were transformed using the Blom formula [35]. The characteristics of the sample are presented as mean values and SDs or percentages. To test baseline differences between the tele-rehabilitation group and control group, we used an independent t-test for continuous variables and χ^2^ test for categorial binomial variables.

The main effects of the tele-rehabilitation program were tested with two models of analysis of covariance (ANCOVA). In Model 1, we used post-rehabilitation outcomes as dependent variables, group (i.e., tele-rehabilitation rehabilitation vs. control) as a fixed factor and baseline outcomes as a covariate. In Model 2, we additionally performed a sensitivity analysis to test the influence of potential confounders in the results, such as age, sex, educational level, health status, duration of the hospital stay, falls in the last year, and type of fracture. Baseline age, sex, and type of fracture were the only variables that demonstrated an additional predictive capacity to Model 1 and were included as covariables in Model 2 in addition to the baseline outcomes.

The z-scores for each outcome at post-rehabilitation were also formed by dividing the difference of the post-rehabilitation raw score of each participant from the baseline mean by the baseline standard deviation (i.e., (post-rehabilitation individual raw value baseline mean)/baseline SD). This way of reporting the effects has been used in recent leading RCTs [36] and has two main advantages: (1) provides standardized estimates that allow comparisons among outcomes with different original measurement units, and (2) these z-scores of change can be interpreted as effect size indicating the within-group and between-group changes in standard deviations, e.g., 0.5 z-score means that the mean value at post-rehabilitation is 0.5 SDs higher than the mean value at baseline. Additionally, we calculated Cohen’s d according to the between-subject design [37]. This effect size indicator can be interpreted according to the standard benchmarks, i.e., a value around 0.2 is considered a small effect size, 0.5 is considered a medium effect size, and 0.8 is considered a large effect size [38].

All analyses were performed using the SPSS software (version 25.0, IBM Corporation, Armonk, NY, USA), and the level of significance was set at *p* < 0.05.

## 3. Results

**Figure 1:**
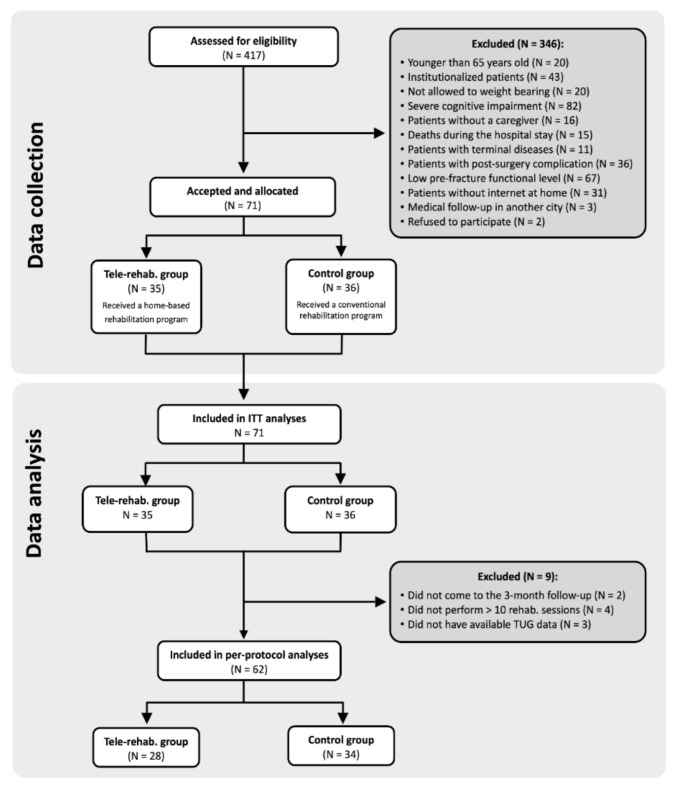
Flowchart with the inclusion/exclusion of participants.

We identified 417 potentially eligible older patients admitted to the hospital for hip fracture, of which 71 participants enrolled in the study and were allocated into the control (N = 36) or tele-rehabilitation (N = 35) groups. A total of 62 participants (28 in the tele-rehabilitation group and 34 in the control group) were included in the per-protocol analysis, while all the 71 participants were included in the intention-to-treat analysis. The reasons for exclusion and dropouts of participants during the data collection and per-protocol analysis are provided in the CONSORT 2010 flow diagram (Figure 1). There were no adverse effects nor deaths reported in either group during the rehabilitation process.

The characteristics of the participants and dividing the sample into both tele-rehabilitation and control groups are shown in Table 1. The only baseline difference was in the age of participants, with the tele-rehabilitation group being close to 4 years younger than the control group (*p* = 0.003), and in the TUG performance, the tele-rehabilitation group having a better performance (*p* = 0.027).

**Figure 2:**
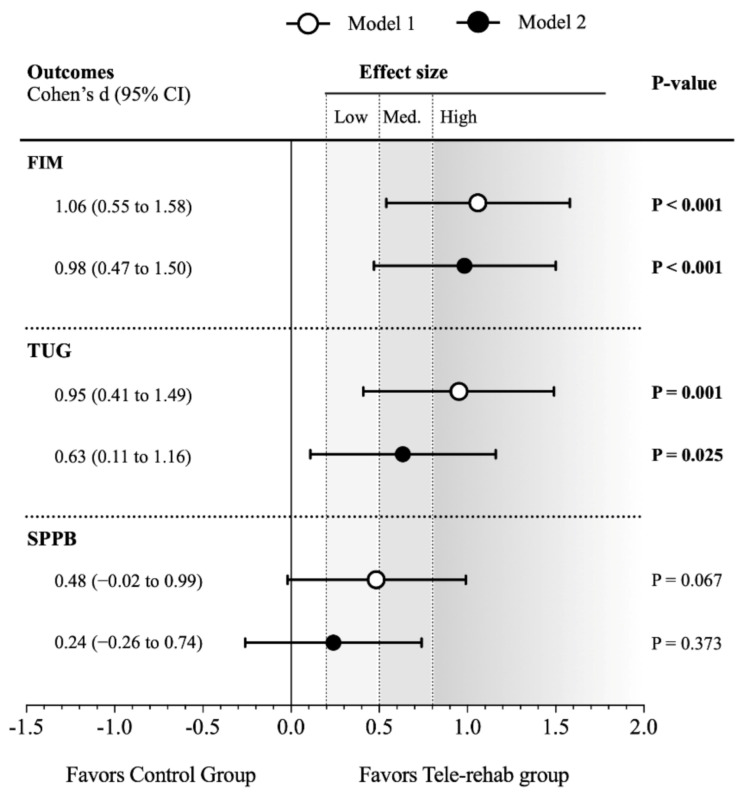
Effect sizes of the ActiveHip project on functional independence and physical performance in the per-protocol analysis.

Table 2 presents the differences between the tele-rehabilitation and control groups 3 months after the hip fracture occurred, adjusting only for baseline values (Model 1) and additionally for age, sex, and type of fracture (Model 2). The total score of the FIM test increased more in the tele-rehabilitation group than in the control group (high effect size: 1.06 Cohen’s d; *p* < 0.001), and this result remained similar in Model 2 (high effect size: 0.98 Cohen’s d; *p* < 0.001). Regarding the physical function evaluated through the TUG, the tele-rehabilitation group had a greater decrease in performance time in comparison with the control group (high effect size: 0.95 Cohen’s d; *p* = 0.001), and this effect was slightly attenuated in Model 2 (medium effect size: 0.63 Cohen’s d; *p* = 0.025). Lastly, the tele-rehabilitation group had a better improvement in the SPPB score than the control group, although this difference was not statistically significant in neither Model 1 (0.48 Cohen’s d; *p* = 0.067) and Model 2 (0.24 Cohen’s d; *p* = 0.373). All these results are graphically presented in Figure 2.

**Figure A1:**
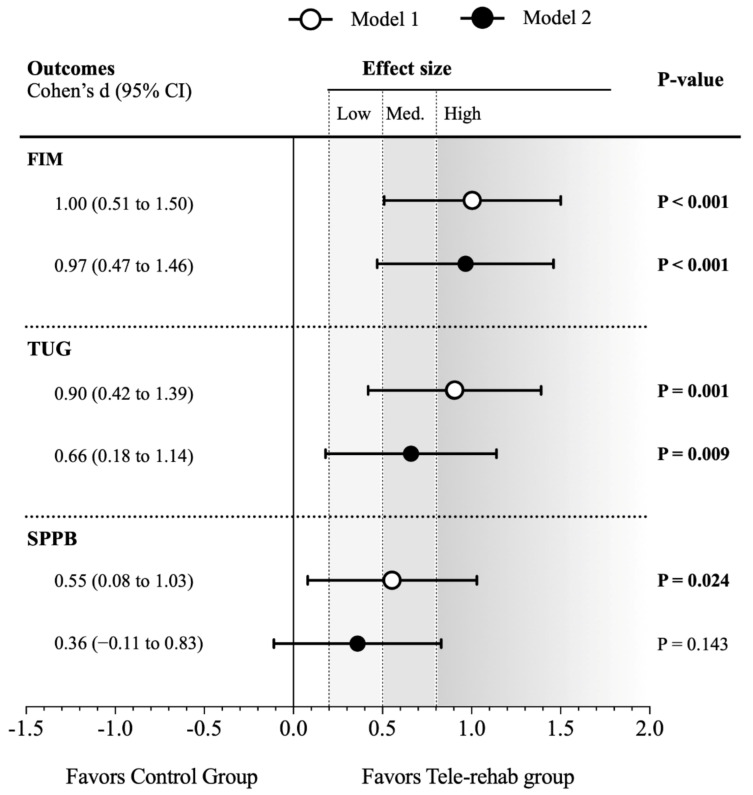
Effect sizes of the ActiveHip project on functional independence and physical performance in the intention-to-treat analysis.

The intention-to-treat represents the secondary analysis and is shown in Appendix A (Table A1, Table A2 and Figure A1). Overall, effects size was slightly attenuated with respect to the per-protocol analysis in the FIM and TUG results. Unlike the per-protocol analysis, the tele-rehabilitation showed a significantly better recovery of the SPPB overall score in comparison with the control group in Model 1 (medium effect size: 0.55 Cohen’s d; *p* = 0.024), although the significance disappeared in Model 2 (0.35 Cohen’s d; *p* = 0.143).

## 4. Discussion

This study compared the effects of a novel 12-week tele-rehabilitation program (delivered by caregivers) against a home-based in-person rehabilitation (delivered by health providers) on functional outcomes of older adults that suffered a hip fracture. Regardless of the intervention received, both groups improved function, but the tele-rehabilitation program was superior at three months for improving function, as measured by the FIM and TUG test.

In this study, we noted that the tele-rehabilitation was superior to a more traditional home care rehabilitation. These results extend the limited evidence for tele-rehabilitation for this population [12,13,15]. However, despite the statistically significant changes favoring the tele-rehabilitation program at the 3-month follow-up on FIM and TUG [34], we recommend caution when interpreting these results because of the study design: choice-based non-randomized group allocation and limited generalizability for the population of interest. Nevertheless, these results are promising since tele-rehabilitation overcame the traditional face-to-face rehabilitation delivered by health providers at home in functional outcomes, such as functional independence and physical performance. This knowledge generates hypotheses to test the tele-rehabilitation program in different sub-populations of older adults with hip fracture, including people living in rural and/or remote regions where health care resources may be scarce.

There are some differences between our study and the few published tele-rehabilitation studies conducted with older adults with hip fracture [13,14,15]. Patients in this study were in the early phase of recovery (first week after hip surgery), while other studies were conducted later: in rehabilitation settings [15], 1–3 months [14], or 5 months after hospital discharge [13]. The shorter period of time between hip surgery and the beginning of our tele-rehabilitation program explains the lower functional level of our patients at baseline compared with other studies [14,15]. However, the patient-reported functional recovery of the patients receiving the @ctivehip program is comparable to previous results observed in a tele-rehabilitation intervention [15]. The @ctivehip program was initiated in the acute phase and supported clinical care guidelines via delivery of an early intervention that extends across the continuum of care.

The older adults included in this study shared some similarities to previous studies for patients with hip fracture, such as the absence of cognitive impairments or the higher proportion of women [13,14,15], although our patients were older than in previous studies [14,15]. This may be because we invited family caregivers to support patients to use the online platform and supervise the exercises at home. Caregiver involvement is a positive predictive factor on patients’ recovery [39]: We acknowledge that it also increases the demands on caregivers, as highlighted previously [40]. Nevertheless, to our knowledge, this is the first tele-rehabilitation program for older adults with hip fracture that gives an active role to caregivers who considered the program as an opportunity to increase their knowledge for patients management at home and enhance their functional recovery [41].

We observed variation for adherence to the tele-rehabilitation program, but in general, it decreased over time. In our study, only 15% of patients completed the full program (50–60 sessions), but 22 patients (63%) completed >20 sessions. Despite the low adherence to the full @ctivehip protocol, patients obtained very good functional recovery (96.8% of their previous pre-fracture FIM). Thus, it is possible that patients did not need as many sessions as were offered. We tried to tailor the intervention by prescribing the program after baseline assessment and re-evaluation at one month (either online or via telephone). But as we did not monitor patients daily [13] or more frequently, we do not know the precise dose for functional recovery. Future studies could test activity monitors and or other feedback and monitoring strategies to support adherence or progression of exercise prescription.

This study has two main strengths to highlight. First, to our knowledge, it is the first tele-rehabilitation program for patients with hip fracture offered during the acute phase and after hospital discharge. Second, the inclusion of caregivers (in both groups) to support patients’ active role during recovery. Nevertheless, the main limitation of the present study is the non-randomization in the allocation of the participants. All statistical analyses were adjusted for baseline values and sensitivity analysis were performed accounting for potential confounders (i.e., age, sex, educational level, health status before the hip fracture, duration of the hospital stay, falls in the last year, and type of fracture) to address that limitation. Choice of group allocation could introduce other sources of bias, such as higher motivation or amount of support from caregivers. However, patients in the control group received in-person rehabilitation at home delivered by an OT or PT, while caregivers supervised the @ctivehip intervention. Thus, there were possible advantages to being in either group. Further, despite the possible bias introduced with choice-based allocation, this study provided the opportunity to appreciate patient preferences and program implementation in the real-world setting to support future clinical decision-making [21]. Further, having the choice of intervention is consistent with a person-centered approach to guide clinical practice [42]. Lastly, a 3-month follow-up period might not be enough to have a whole picture of the rehabilitation process of hip fracture patients, and future intervention trials should include longer follow-up periods.

## 5. Conclusions

This study highlights that for older adults with hip fracture, a 12-week tele-rehabilitation program supervised by family caregivers had better results in functional independence and physical condition (self-report and performance-based) than traditional home-based rehabilitation. These results suggest the use of ICTs could be a management option in the recovery process for patients with hip fracture and their caregivers.

## Figures and Tables

**Table 1:** Baseline characteristics and post-intervention raw values of sample divided by tele-rehabilitation (tele-rehab.) and control group.

Variables	Tele-Rehab. (n = 28)	Control (n = 34)	*p*
Age (years)	75.86 ± 5.79	80.38 ± 5.54	**0.003**
Weight (kg)	68.1 ± 9.94	69.15 ± 10.21	0.708
Height (cm)	160.88 ± 7.32	158.04 ± 8.83	0.215
BMI (kg/m^2^)	26.38 ± 3.98	27.63 ± 3.58	0.248
Gender, n (%)			0.557
Men	8 (28.6%)	9 (26.5%)	
Women	20 (71.4%)	27 (73.5%)	
**Outcomes Basal**			
FIM (18–126 points)	77.75 ± 4.22	78.12 ± 6.61	0.800
TUG (seconds)	66.53 ± 36.89	99.72 ± 68.82	**0.027**
SPPB (0 to 12 points)	3.21 ± 1.17	2.58 ± 1.46	0.072
**Outcomes Post-rehab.**			
FIM (18–126 points)	120.54 ± 7.48	108.29 ± 14.67	
TUG (seconds)	12.95 ± 4.94	24.38 ± 13.56	
SPPB (0 to 12 points)	8.36 ± 2.39	5.94 ± 3.01	
**Confounder**			
Type of fracture			0.123
Intracapsular	15 (53.6%)	11 (32.3%)	
Extracapsular	13 (46.4%)	23 (67.7%)	

SD = standard deviation; n = sample size; FIM: Functional Independence Measure; TUG: Timed up and go; SPPB: Short Physical Performance Battery; Basal: after the hip fracture and before de rehabilitation; Post: after the rehabilitation (3-month follow up). Values are presented as mean ± SD or percentages. For continuous variables, *p*-value was obtained by an independent samples T-test, whereas for categorical variables, *p*-value was obtained by chi-square test. Significant differences (*p* < 0.05) are highlighted in bold.

**Table 2:** Intervention effects of the ActiveHip project.

Statistical Models Outcomes	Tele-Rehabilitation Group		Control Group		Z-Score Differences Tele-Rehab-Control (95% CI)	*p*
	N	Z-Score (95% CI)	N	Z-Score (95% CI)		
**Model 1**						
FIM	30	0.50 (0.18 to 0.82)	35	−0.44 (−0.73 to −0.14)	0.93 (0.49 to 1.37)	**<0.001**
TUG	28	−0.45 (−0.76 to −0.14)	34	0.32 (0.03 to 0.62)	−0.77 (−1.21 to −0.34)	**0.001**
SPPB	30	0.33 (−0.03 to 0.69)	35	−0.15 (−0.5 to 0.2)	0.48 (−0.03 to 0.98)	0.067
**Model 2**						
FIM	30	0.44 (0.13 to 0.75)	35	−0.39 (−0.67 to −0.1)	0.83 (0.40 to 1.25)	**<0.001**
TUG	28	−0.28 (−0.55 to −0.01)	34	0.17 (−0.09 to 0.43)	−0.45 (−0.84 to −0.06)	**0.025**
SPPB	30	0.20 (−0.15 to 0.56)	35	−0.03 (−0.38 to 0.32)	0.24 (−0.29 to 0.76)	0.373

CI = confidence interval; n = sample size; N = Newton. A one-way analysis of covariance (ANCOVA) was used to test z-score differences between the tele-rehabilitation and control group at the post-intervention, adjusting for basic pre-intervention values (Model 1) and additionally for the participants’ sex. age, and the type of hip fracture (Model 2). Adjusted means and confidence intervals of the mean are represented. Differences between groups are presented as post-intervention adjusted mean minus pre-intervention adjusted mean. Significant differences (*p* < 0.05) are highlighted in bold.

## Data Availability

This study did not report any public data.

## Appendix A

**Table A1:** Baseline characteristics of sample divided by tele-rehabilitation (tele-rehab.) and control group.

Variables	Tele-Rehab. (n = 35)	Control (n = 36)	*p*
Age (years)	76.71 ± 6.04	80.72 ± 5.59	**0.005**
Weight (kg)	68.65 ± 9.45	69.05 ± 8.94	0.855
Height (cm)	160.6 ± 6.69	157.61 ± 7.68	0.085
BMI (kg/m^2^)	26.7 ± 3.95	27.82 ± 3.23	0.195
Gender. n (%)			1.000
Men	9 (25.7%)	9 (25.0%)	
Women	26 (74.3%)	27 (75.0%)	
**Outcomes Basal**			
FIM Basal	77.46 ± 5.48	78.22 ± 6.48	0.593
TUG Basal (seconds)	81.02 ± 71.04	99.37 ± 63.71	0.256
SPPB Basal	3.03 ± 1.32	2.58 ± 1.36	0.166
**Outcomes Post-rehab.**			
FIM Post	119.23 ± 8.15	108.5 ± 14.45	
TUG Post (seconds)	15.05 ± 9.01	23.96 ± 13.38	
SPPB Post	7.86 ± 2.99	6.00 ± 2.98	
**Confounder**			
Type of fracture			0.232
Intracapsular	17 (48.6%)	12 (33.3%)	
Extracapsular	18 (51.4%)	24 (66.7%)	

SD = standard deviation; n = sample size; FIM: Functional Independence Measure; TUG: Timed up and go; SPPB: Short Physical Performance Battery; Basal: after the hip fracture and before de rehabilitation; Post: after the rehabilitation (3-month follow up). Values are presented as mean ± SD or percentages. For continuous variables. *p*-value was obtained by an independent samples T-test. Whereas for categorical variables, the *p*-value was obtained by the chi-square test. Significant differences (*p* < 0.05) are highlighted in bold.

**Table A2:** Intervention effects of the ActiveHip project.

Statistical Models Outcomes	Tele-Rehabilitation Group		Control Group		Z-Score Differences Rehab–Control (95% CI)	*p*
	N	Z-Score (95% CI)	N	Z-Score (95% CI)		
**Model 1**						
FIM	35	0.45 (0.15 to 0.75)	36	−0.45 (−0.74 to −0.15)	0.89 (0.47 to 1.32)	**<0.001**
TUG	35	−0.37 (−0.65 to −0.10)	36	0.36 (0.09 to 0.64)	−0.74 (−1.13 to −0.35)	**<0.001**
SPPB	35	0.30 (−0.04 to 0.64)	36	−0.26 (−0.59 to 0.08)	0.55 (0.08 to 1.03)	**0.024**
**Model 2**						
FIM	35	0.41 (0.12 to 0.70)	36	−0.41 (−0.70 to −0.13)	0.82 (0.42 to 1.23)	**<0.001**
TUG	35	−0.24 (−0.48 to 0.00)	36	0.24 (−0.01 to 0.47)	−0.48 (−0.83 to −0.12)	**0.009**
SPPB	35	0.19 (−0.13 to 0.52)	36	−0.16 (−0.48 to 0.17)	0.35 (−0.12 to 0.82)	0.143

CI = confidence interval; n = sample size; N = Newton. A one-way analysis of covariance (ANCOVA) was used to test z-score differences between the intervention and control group at the post-intervention, adjusting for basic pre-intervention values (Model 1) and additionally for the participants’ sex. age, and the type of hip fracture (Model 2). Adjusted means and confidence intervals of the mean are represented. Differences between groups are presented as post-intervention mean minus pre-intervention mean. Significant differences (*p* < 0.05) are highlighted in bold.
